# miR302 regulates SNAI1 expression to control mesangial cell plasticity

**DOI:** 10.1038/srep42407

**Published:** 2017-02-14

**Authors:** L. De Chiara, D. Andrews, A. Watson, G. Oliviero, G. Cagney, J. Crean

**Affiliations:** 1 UCD Diabetes Complications Research Centre, Conway Institute of Biomolecular and Biomedical Science, Dublin, Ireland; 2 UCD School of Biomolecular and Biomedical Science, University College Dublin, Belfield, Dublin, Ireland; 3 Weill Cornell Medical College (WCMC), Department of Surgery, 1300 York Avenue, 10065 New York (NY), USA; 4 Syddansk Universitet - Odense Universitet Institut for Biokemi og Molekylær Biologi, Danmark

## Abstract

Cell fate decisions are controlled by the interplay of transcription factors and epigenetic modifiers, which together determine cellular identity. Here we elaborate on the role of miR302 in the regulation of cell plasticity. Overexpression of miR302 effected silencing of the TGFβ type II receptor and facilitated plasticity in a manner distinct from pluripotency, characterized by increased expression of Snail. miR302 overexpressing mesangial cells also exhibited enhanced expression of EZH2 coincident with Snail upregulation. esiRNA silencing of each component suggest that Smad3 and EZH2 are part of a complex that regulates plasticity and that miR302 regulates EZH2 and Snail independently. Subsequent manipulation of miR302 overexpressing cells demonstrated the potential of using this approach for reprogramming as evidenced by *de novo* expression of the tight junction components ZO-1 and E-cadherin and the formation of ZO-1 containing tight junctions. Understanding the processes through which dynamic epigenetic silencing is controlled in adults cells will allow us to address the epigenetic state of acquired disease and whether original states, regenerative in nature, can be restored with therapy.

Diabetes mellitus is a complex metabolic disorder, the 5^th^ leading cause of mortality worldwide resulting in more than 4 million deaths annually. Recent reports have predicted a 150% increase in occurrence in the next 20 years, with a major burden on medicinal care due to its devastating complications^1^. Diabetic nephropathy (DN) is a common complication of diabetes, with 25–45% of patients developing renal fibrosis and progressing to end stage renal disease^2^. There is no cure for DN and therapeutic efforts are focused on limiting loss of renal function and associated symptoms^3^. During the last fifteen years, significant advances have been made concerning the mechanisms underlying initiation and progression of chronic kidney disease. The capacity of renal mesangial cells to undergo remodelling and acquire fibroblastic plasticity was first suggested by studies from our laboratory that identified the recapitulation of ontogenic gene expression profiles in experimental models of diabetic nephropathy and in patients^4^ (Supplementary Fig. S1). Subsequent studies have extensively characterised the role of Transforming Growth Factor β1 (TGFβ1) in mediating these change however despite significant efforts in this area, therapeutic interventions have yet to demonstrate clinical efficacy. New paradigms are emerging from recent studies elucidating the instructive role of TGFβ during embryonic development, coupled with the identification of parallel processes in adult tissues^5^.

Cell fate specification is a progressive process of diversification through which a cell, by undergoing profound changes in gene expression and regulation, takes its role within a defined context. On the other hand, cell fate conversion is considered a process by which a cell can change its phenotype and acquire a new and distinct “altered” fate^6^. While the first process is pivotal during development, the latter is increasingly recognized as fundamental not only during embryogenesis but also in numerous disease states^7^,^8^. A cell must acquire a plastic phenotype in order to properly adapt and respond to environmental stimuli. These adapting processes involve and are controlled by the interplay between microRNAs, transcription factors (TFs), and epigenetic modifiers that work in concert to determine cell fate.

Human Mesangial Cells (HMCs) are a specialized type of microvascular pericyte^9^ anchored to the glomerular membrane. Due to their intrinsic nature, these cells are highly plastic and responsive to the surrounding microenvironment. These responsive mechanisms often result in detrimental processes being triggered by extracellular stimuli that can lead to the destruction of the complex glomerular and renal ultrastructure^10^. Frequently, these alterations result in a change in cytoskeletal-mediated contractility, reflected in dynamic focal adhesions^11^. Perhaps this is best evidenced by the apparent alterations in actin dynamics mediated by TGFβ and CTGF, reflecting changes in contractility both *in vivo* and *in vitro*; previous studies carried out in our laboratory have demonstrated that HMCs acquire plasticity triggered by hyperglycaemia and growth factors^4^ which greatly enhances the expression of a family of microRNAs, the miR302 family. The miR302 family is composed of 4 members, miR302a/b/c/d that are transcribed as a single polycistronic cluster^12^; this cluster is prominently expressed in Embryonic Stem Cells (ESCs) while its expression is decreased during differentiation and commitment^13^. It has been previously shown that miR302 can promote iPSC (induced Pluripotent Stem Cells) generation^14^,^15^,^16^ and its expression is directly regulated by the stemness factors, Oct4 and Sox2, and Nanog^17^,^18^.

The primary validated target of miR-302 is the Transforming Growth Factor (TGF)-β Type II receptor (TβRII)^14^. TGFβ has a prominent role in triggering Epithelial to Mesenchymal Transition (EMT) during embryogenesis and activating parallel processes in disease^19^. Strikingly, miR302 is able to control and impact on the TGFβ pathway in an extensive and context-dependent manner; it has a well-established role in promoting the acquisition of pluripotency by targeting the TβRII, thus blocking the activation of the pathway^12^,^14^, however it can also propagate and promote its activation in ESCs by levelling the expression of LEFTY1^20^.

The identification of embryonic stem cell specific miRNAs led to the widely accepted hypothesis that interplay between specific microRNAs and their repressed targets controls both the maintenance of stemness and the specification of cell types^21^,^22^. Increasingly, parallel processes in pathogenesis are recognised as critical mediators of damage and repair. Specifically, the potential role of the miR302 and Let-7 families in both these processes have been recently established by our group and others^4^,^23^.

In addition to these processes, a third level of regulation, involving the remodelling of the chromatin environment, has emerged as numerous studies have demonstrated that cell type specific regulatory genes can be identified by specific histone marks^24^. Among others, changes in the methylation status of histone H3 have been associated with stemness, cell specification and numerous diseases^25^,^26^. The methylation of histone H3 on the lysine 4 (H3K4) and and 26 (H3K26) are generally associated with active transcription^27^, whereas permissive promoters are enriched with both active (H3K4) and repressive marks (H3K27) and considered to exist in a “poised” state^27^,^28^. Central to these processes is the Polycomb Repressive Complex 2 (PRC2), which contains EZH2 (Enhancer of zeste homolog 2), a histone methyltransferase that catalyzes the trimethylation of H3K27^29^, mediating gene repression, and additional core components EED, SUZ12 and RBBP4/RbAp48/NURF55.

In the present study we investigated the role of miR302 in regulating mesangial plasticity and explore the idea that partial reprogramming of mesenchymal cells leads to the acquisition of a “poised” state that may be manipulated for therapeutic repair.

## Results

### Overexpression of miR302a/b/c/d in Human Mesangial Cells

**Figure 1:**
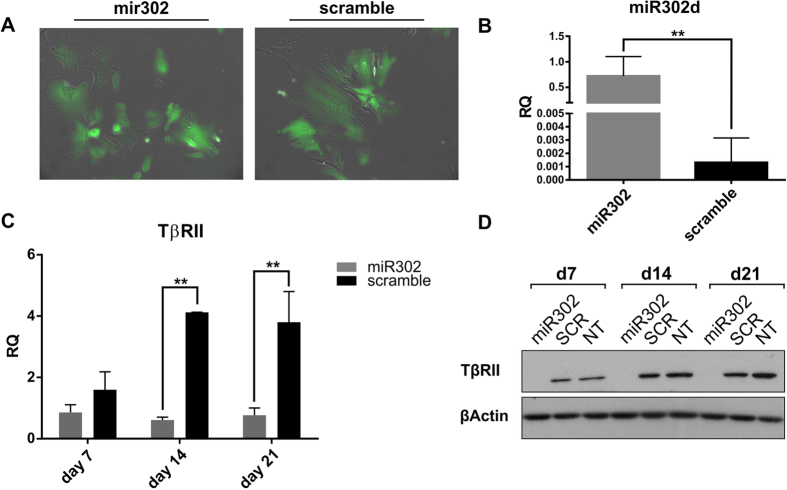
Overexpression of miR302a/b/c/d in Human Mesangial Cells silences the TGFβ type II receptor. (**A**) Representative images of GFP^+^ HMCs 7 days post transduction with miR302 (left panel) and scramble virus (right panel). Original magnification x100. 7 days post transduction, HMCs showed a marked upregulation of miR302d expression when compared to scramble infected cells (**B**). As a result of miR302 overexpression TβRII expression is decreased at mRNA (**C**) and protein (**D**) level, across all the analysed time points. Graph B and C and panel (**D**) are representative of 3 independent experiments. (SCR: scramble; NT: Non-treated cells; GFP: Green Fluorescent Protein; TβRII: TGFβ Receptor II; RQ: Relative Quantification normalized on 18 S for TβRII or RNU6B for miR302d; **P < 0.01).

HMCs were seeded at a very low confluency and then incubated for 48 h with a polycistronic lentiviral vector encoding all four members of the miR302 family and a Green Fluorescent Protein (GFP)-reporter. At 7 days post transduction all cells demonstrated clear GFP expression (Fig. 1A). The expression of the miR302d was analysed by RealTime PCR as a readout of the level of expression of miR302 in the cells. 7 days post lentiviral transduction, a marked increase in miR302d expression (Fig. 1B) was observed, indicating successful transduction. RNA and protein were extracted at various time points in order to investigate the phenotypic changes caused by the miR302 overexpression system. One of the best-characterised targets of the miR302 family is the TβRII^4^,^14^. TβRII is involved in EMT and its activation leads to the phosphorylation of Smad2 and Smad3 resulting in their translocation from the cytoplasm to the nucleus^30^. We verified the effective downregulation of TβRII by both RNA (Fig. 1C) and protein analysis (Fig. 1D). As expected, the receptor is repressed throughout all time points. miR302 is the most important and abundant microRNA present in human ESCs (hESCs)^12^. Since its promoter can be directly bound and regulated by Oct4^17^,^18^ and various reports have highlighted its ability to induce Oct4 expression^16^, we investigated whether miR302 overexpression caused the acquisition of a pluripotent phenotype in HMCs. RealTime PCR analysis for Oct4 and Nanog was performed at various time points (Supplementary Fig. S2) showing no expression of either transcription factors. Noticing the appearance of rounded granulated colonies between 14 and 21 days post-transduction, we hypothesised that these colonies originated from HMCs cells successfully reprogrammed toward pluripotency. After picking, HMC-derived colonies were cultured on matrigel under stem cell-like conditions for up to 21 days. These colonies were able to attach to the coated plates and proliferate. Although they became bigger and tried to divide (Supplementary Fig. S3A), no obvious hallmarks of a pluripotent phenotype were observed nor was there any significant change in Oct4 expression (Supplementary Fig. S3B). Taken together, these results demonstrated the successful transduction of HMCs with miR302 lentivirus and its ability to block TβRII expression. Moreover, they showed that miR302 upregulation alone is not sufficient to reprogram HMCs to pluripotency.

### miR302 upregulates Snail expression in HMCs

**Figure 2:**
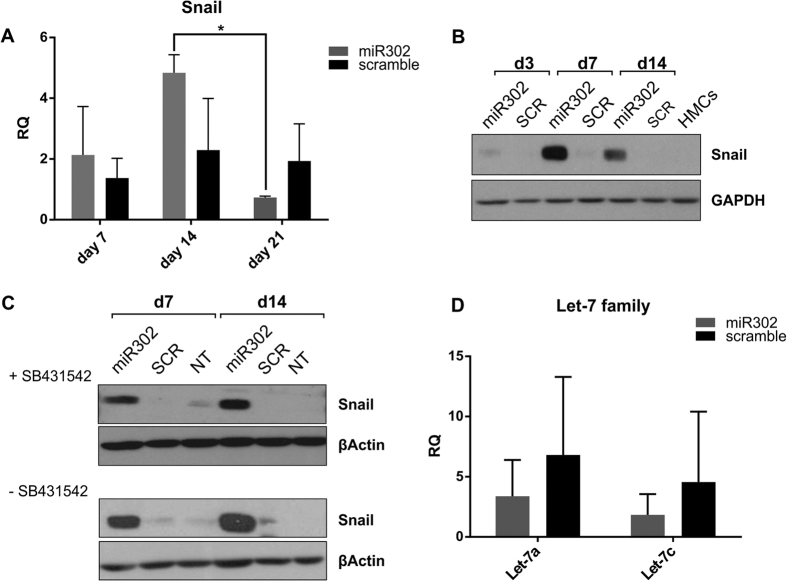
miR302 increases the expression of Snail during the acquisition of plasticity. (**A**) Demonstrates increased expression of Snail in miR302-HMCs compared to scramble HMCs. During the first week following miR302 overexpression in HMCs, Snail is upregulated at both mRNA (**A**) and protein level (**B**). Snail expression is not blocked by the addition to the culture media of the SB431545 (**C**), a potent inhibitor of TGFβ Receptor I (top panel) compared to standard culture conditions (bottom panel). Given the reciprocal nature of miR302 and Let-7 family expression and considering that Snail has been recently reported to bind and repress the promoter of the Let-7 family we analysed the level of expression of two of the components of this family, Let-7a and Let-7c, noticing no difference 7 days post transduction (**D**). All the figures are representative of 3 independent experiments. (*P < 0.05).

Having verified the lack of pluripotency in miR302-HMCs, we proceeded to analyse the phenotype acquired by the cells. Interestingly, increased expression of Snail (or SNAI1) was consistently observed at 3 days and 7 days post transduction in miR302 overexpressing cells, although some variability in levels were apparent, likely reflecting the heterogeneous and asynchronous nature of the cell populations at these time points. (Figure 2A,B, quantified in Supplementary Fig. S4A). We verified that this upregulation was not due to a nonspecific effect by analysing the resulting Snail expression in an additional arbitrarily chosen cell type (Supplementary Fig. S5A) after miR302 overexpression. This was a particularly unexpected result as Snail is widely regarded as the most important transcription factor involved in driving EMT as a result of TβRII/TβRI activation. No increased expression of Snail was apparent in HMCs following TGFβ treatment (Supplementary Fig. 5B) and additionally, they also exhibited downregulation of miR302 (Supplementary Fig. 5C), supporting the idea that Snail is not regulated by TGFβ in this context.

To rule out the possibility that Snail expression was linked to an alternative mechanism of activation of the TGFβ Receptor I (TβRI) we treated miR302-HMCs with a potent and specific inhibitor of the receptor^31^, SB431543, 7 days post miR302 transduction. After 7 and 14 days of treatment with the inhibitor (Fig. 2C, quantified in Supplementary Fig. S4B,C) the cells maintained a high level of Snail protein expression, comparable to the standard culture condition (Fig. 2C). Similarly, Snail mRNA expression is maintained throughout the 2 weeks of treatment with the inhibitor (Supplementary Fig. S4D). A recent study from the Daley laboratory demonstrated that Snail is paradoxically required in order to successfully reprogram fibroblasts to pluripotency^32^. They showed how Snail promotes the acquisition of plasticity by binding the promoter of the Let-7 family of microRNAs, thus causing its downregulation. This hypothesis is particularly fascinating when considering the reciprocal relationship that occurs between miR302 and Let-7^13^. In order to verify whether this mechanism is valid in this context, we measured the levels of two members of the Let-7 family, Let-7a and Let-7c, 7 days post transduction. Although the levels were lower in miR302 cells when compared to scramble cells, no statistically significant difference was detected (Fig. 2D), demonstrating that Snail in this context does not suppress Let-7 expression.

These results demonstrated, for the first time, that miR302 drives Snail expression in HMCs, during the acquisition of plasticity.

### EZH2 upregulation in miR302-HMCs is independent from Snail activation

**Figure 3:**
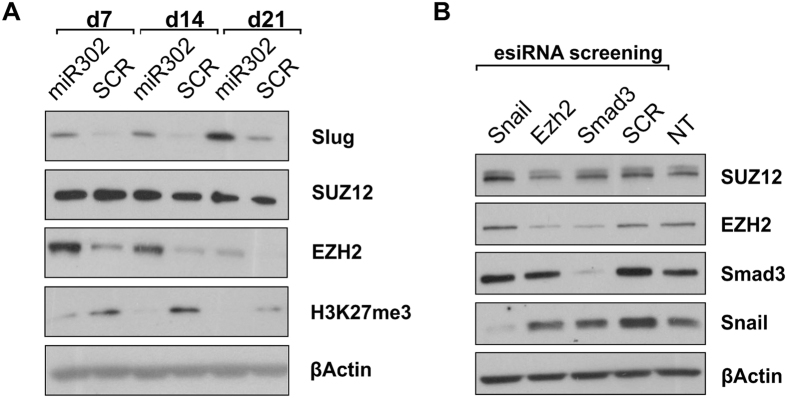
miR302 upregulates EZH2 independently from Snail expression, whereas Smad3 and EZH2 demonstrate co-dependence. The expression of the PRC2 components was assessed at days 7, 14 and 21 post transduction (**A**). No changes were observed in SUZ12 across all the time points whereas EZH2 follows the same pattern as both Snail and Slug, another member of the Snail family. Of note, the trimethylation of lysine 27 of histone H3, which is readout of the EZH2 activity, is unexpectedly turned off. EZH2, Snail and Smad3 were knocked down using esiRNA technology in miR302-HMCs, demonstrating that EZH2 and Snail are independently upregulated by miR302 overexpression in HMCs (**B**). In contrast the Smad3 knock down results in decreased expression of EZH2 suggesting cooperativity between EZH2 expression and Smad3 (**B**). (NT: non-transfected cells; H3K27me3: trimethylation of the lysine 27 on the histone H3). All panels are representative of 3 independent experiments.

As Snail plays a role in enhancing the acquisition of pluripotency in fibroblasts^32^ and pathogenic plasticity during renal failure^33^, we analysed the expression of other proteins known to be involved in these processes. We verified that Slug (or SNAI2), another member of the Snail family is similarly upregulated within the first 2 weeks post transduction (Fig. 3A, quantified in Supplementary Fig. S6A). Intriguingly, miR302-HMCs also exhibited enhanced expression of EZH2 coincident with Snail upregulation. EZH2 is the catalytic subunit of the PRC2 Complex and it catalyses the trimethylation of the lysine 27 on histone H3^34^; by doing so the PRC2 complex can repress gene transcription through the control of promoter access^29^. EZH2 has been widely studied as a protein implicated in cancer and metastatic progression and its upregulation is linked to an aggressive phenotype and to enhanced proliferation by cancer cells^35^. Interestingly, in miR302-HMCs, EZH2 showed the same expression pattern as Snail, being significantly upregulated within the first 2 weeks post transduction, while its expression subsequently decreases (Fig. 3A, quantified in Supplementary Fig. S6A). Of note, the H3K27 tri-methylation mark, a repressive mark produced by EZH2 activity, was unexpectedly decreased, irrespective of EZH2 upregulation. Moreover, no difference was detected across all the time points for SUZ12, another core component of the PRC2 complex (Fig. 3A, quantified in Supplementary Fig. S6A), suggesting a specific effect on EZH2 by miR302. Finally, we established the correct localization in the nuclear fraction of EZH2 and Snail (Supplementary Fig. S7). Interestingly, in miR302-HMCs the downregulation of TβRII by miR302 overexpression did not affect the localisation of Smad3 in the nuclei (Supplementary Fig. S7), suggesting again a TGFβ independent effect of miR302 in HMCs. A recent paper from the Liang group, demonstrated that Snail expression in cancer cells is able to upregulate EZH2 by inhibiting miR101 expression^36^. As EZH2 and Snail expression appeared to be tightly linked, and taking into account the fact that Smad3 was still present regardless of the silencing of the TGFβ type II receptor, we decided to knock down EZH2, Snail and Smad3 in miR302-HMCs using esiRNA. At 7 days post transduction, when the HMCs showed a high level of expression of both EZH2 and Snail we knocked down Snail, EZH2 and Smad3 respectively, while a scrambled off-target sequence was used as a control. Interestingly, only the Smad3 knockdown resulted in the downregulation of EZH2 suggesting that the 2 proteins may form a cooperative, regulatory complex. Knock down of EZH2 does not affect Snail and vice versa (Fig. 3B). SUZ12 was used as an unrelated control for off-target effect. Taken together, these results demonstrated that miR302 regulates EZH2 and Snail expression independently.

### miR302-HMCs do not acquire a cancer-like phenotype

**Figure 4:**
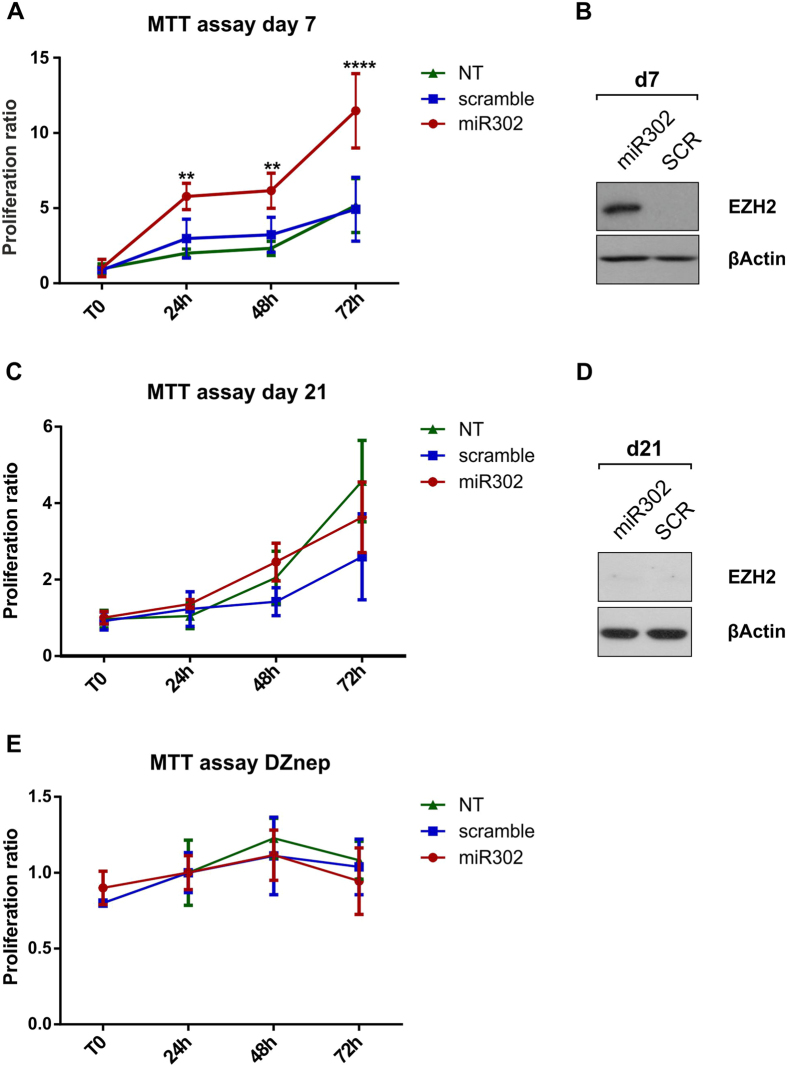
EZH2 expression correlates with increased proliferation of miR302-HMCs. mir302, scramble and non-transduced HMCs were seeded at a low confluency and an MTT assay was performed at 7 days (**A**) and 21 days (**B**) post transduction. Increased proliferation was observed 7 days post transduction correlating with the level of expression of EZH2 (**B**), while the proliferative advantage of miR302-HMCs is lost (**C**) when EZH2 expression decreases (**D**). This was confirmed by treating the cells with DZnep at the time of seeding (**E**); by ablating the activity of EZH2, the cells are no longer able to proliferate. All data are representative of 4 independent experiments performed separately. (DZnep: 3-Deazaneplanocin A, inhibitor of the EZH2 catalytic activity; **P < 0.01; ***P < 0.001).

Since cancer cells expressing high levels of EZH2 display a higher rate of proliferation^37^,^38^ we performed an MTT assay on miR302-HMCs. A first set of experiments was carried out by seeding miR302-HMCs at 7 days post transduction (Fig. 4A,B) finding, as expected, an increased rate of cell proliferation. Importantly, miR302 cells return to a normal proliferation rate once the expression of EZH2 started to drop (Fig. 4C,D). In order to understand whether the increased proliferation of HMCs was linked to EZH2 activity we treated the cells at the time of seeding with DZnep, an inhibitor of EZH2 catalytic activity^39^. By blocking the activity of EZH2 the cells no longer demonstrated any changes in proliferation (Fig. 4E).

**Figure 5:**
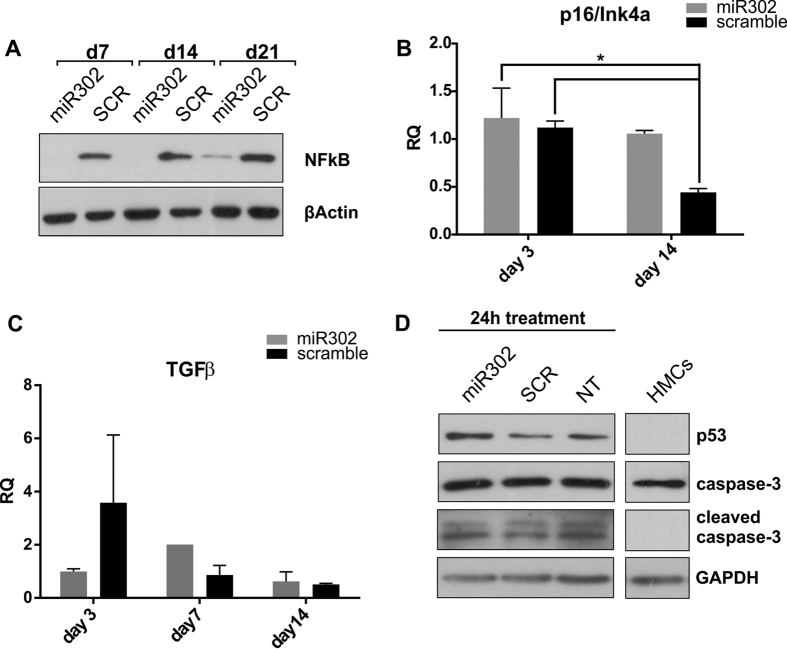
miR302-HMCs acquire plasticity without gaining a tumorigenic phenotype. Typical markers of cancer have been investigated in order to gain more insight into miR302-HMCs phenotype. NFκB, a potent driver of carcinogenesis and inflammation, is dramatically downregulated in miR302-HMCs (**A**), while p16, a well-known tumour suppressor protein is maintained in miR302-HMCs (**B**). Despite the upregulation of Snail, no TGFβ increase is detected at any of the time points analysed (**C**). To test whether miR302-HMCs were protected against apoptosis, we treated the cells with 50 μM of etoposide for 24 h (**D**). No difference is detected in the expression of p53 and the cleaved form of caspase 3 in miR302, scramble and non-treated cells. (*P < 0.05). All panels are representative of 3 independent experiments.

Increased proliferation, together with upregulation of both EZH2 and Snail are accepted hallmarks of tumourigenesis and metastastic progression^36^, so we therefore investigated whether HMCs transduced with miR302 acquired a cancer-like phenotype. A well-known transcription factor involved in tumour progression is NFκB. NFκB is interesting for a number of different reasons; it represents not only an important link between cancer and inflammation^40^ but it is also known for its role as Snail activator promoting cancer aggressiveness^41^. Intriguingly, analysis of NFκB expression by Western Blot (Fig. 5A, quantified in Supplementary Fig. S8), found that it is decrased in miR302-HMCs compared to scramble transduced HMCs. Moreover, no change in TGFβ expression was detected at any of the analysed time points (Fig. 5C).

In hESCs, miR302 inhibits tumorigenicity by controlling G_1_-S cell cycle transition and promoting p16/Ink4a upregulation^42^, a well-known tumour suppressor protein^43^. By doing so, the members of this family protect the pluripotent nature of hESCs, preventing them from giving rise to cancer formation. We analysed p16/Ink4a expression by RealTime PCR (Fig. 5B) and found, in line with the literature, that miR302 overexpression caused an upregulation of p16/Ink4a expression.

Finally, we investigated the involvement of miR302 in promoting/preventing apoptosis. miR302 has been shown to cause apoptosis in cancer cells^44^, while in normal cells it does not affect the apoptotic pathway^42^. To stimulate the activation of the apoptotic pathway in HMCs we treated the cells with etoposide, a potent anticancer drug capable of initiating a program of apoptosis^45^. As shown in in Fig. 5D, no difference was detected among the miR302, scramble and non-transduced groups regarding the appearance of the cleaved form of the caspase 3, while p53 upregulation is slightly diminished in miR302 and scramble cells compared to the non-transduced ones. This consistency, among the different groups, reflects the lack of an aggressive phenotype in miR302-HMCs.

These results are particularly important as they confirm that, although miR302-HMCs acquire a more-plastic phenotype, this process, in healthy cells, is not linked to a cancer-like state, raising the possibility that it can be used to manipulate cell fate and identity.

### miR302-HMCs acquire plasticity that facilitates *de novo* assembly of ZO-1 containing junctions

Taking these results together, miR302-HMCs exhibit a higher degree of plasticity, evidenced by Snail and EZH2 expression and their higher proliferation rate, when compared to scramble or control (non-transduced) HMCs.

**Figure 6:**
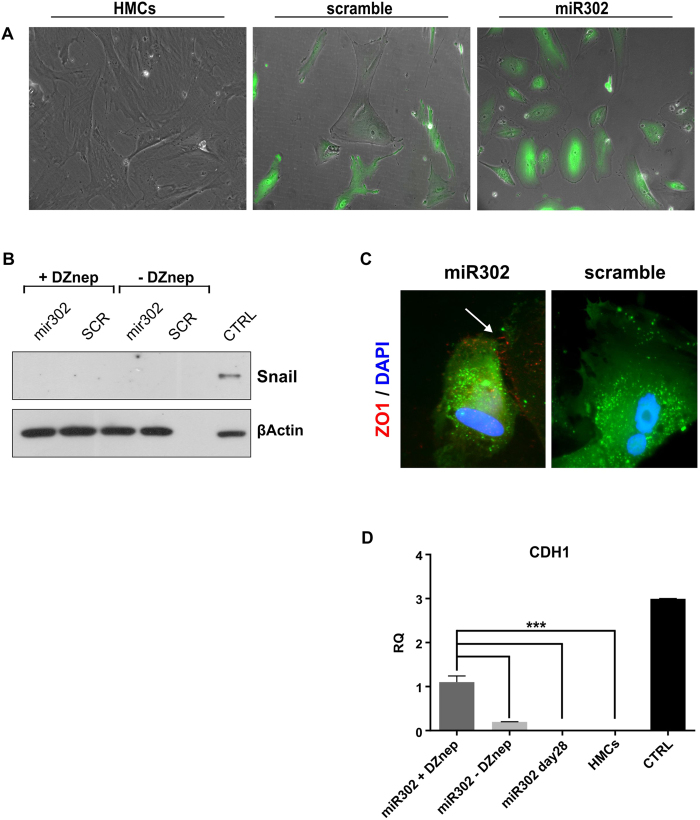
miR302-HMCs form ZO-1 containing junctions when cultured on matrigel. (**A**) Representative picture of normal, scramble and miR302 cells plated on matrigel. Original magnification x100. Mir302-HMCs show *de novo* expression of ZO-1 protein (**C**, white arrow), while they lose the expression of Snail, a marker of mesenchymal cells (**B**). (CTRL: control, HKC8s treated with TGFβ for 24 hrs). Furthermore they show upregulation of CDH1 (E-cadherin) expression at mRNA level (**D**), in particular when treated with DZnep (CTRL: control, HKC8). Original magnification x630. (DAPI: 4′,6-diamidino-2-phenylindole). (***P < 0.001).

In order to evaluate if this “positive” plasticity can be manipulated to push the cells toward a different, more epithelial phenotype, we devised a protocol employing a combination of two different inhibitors of GSK-3β and of the EZH2 activity respectively. DZnep was chosen as previous experiments carried out in our laboratory demonstrated a facility to increase E-cadherin expression in epithelial cells (Supplementary Fig. S9)^46^. Moreover, our group has demonstrated a role for EZH2 in supressing E-cadherin expression in the presence of Smad3^46^ (Manuscript under submission). Similarly, GSK-3β inhibition has been shown to stabilize epithelial junctions making ESCs more “epithelial”^47^. 12 days post miR302 transduction, when the cells exhibit the highest level of Snail and EZH2 expression, HMCs were plated on matrigel with the addition of the inhibitors. Within days of switching to a different microenvironment, miR302-HMCs started to acquire a more cobblestoned shape, (Fig. 6A), while scramble cells stopped proliferating and died. To assess the nature of these cobblestoned cells we stained the miR302-HMCs for Zonula Occludens (ZO)-1, a protein contained in the tight junction. The miR302-HMCs, demonstrated *de novo* tight junction formation (white arrows, Fig. 6C), indicating that the cells have lost their characteristic scattered phenotype and formed connections with the surrounding cells. Interestingly, expression of the transcriptional repressor Snail was silenced in both miR302 and scramble cells after plating on matrigel (Fig. 6B). Finally, miR302-HMCs showed a statistically relevant upregulation of CDH1 (E-cadherin) expression at mRNA level (Fig. 6D), in particular when treated with DZnep; overexpression of miR302 alone is not sufficient to trigger CDH1 expression.

These results demonstrated for the first time the *de novo* expression of epithelial junctions components by human mesangial cells demonstrating the potential of utilising such an approach for reprogramming in chronic renal disease.

## Discussion

In the context of chronic renal diseases, TGFβ plays an important role in directing cellular damage in the DN milieu^48^. The TGFβ superfamily is composed of activins, nodal, Bone Morphogenetic Proteins (BMPs), Growth and Differentiation Factors (GDFs) and anti-Müllerian hormone (AMH). They act by regulating various developmental and homeostatic processes and are involved in numerous human diseases^49^ as well as being considered a viable therapeutic target; nevertheless their pleiotropic signalling has resulted in limited success in targeting their activity. New insights provided herein suggest that miR302 plays a critical role in regulating epigenetic phenomena that control cell fate.

Diabetes mellitus is a complex metabolic disorder characterised by persistent hyperglycaemia; emerging evidence indicates that multiple factors involved in the aetiology of diabetes can alter epigenetic mechanisms and regulate susceptibility to microvascular complications. Particularly important is the role of the PRC2 methyltransferase, EZH2. EZH2 is the catalytic subunit of the PRC2 complex which catalyses the trimethylation of lysine 27 on the histone H3 and mediates genes silencing^29^. It is involved in repressing the E-cadherin promoter during EMT and its expression correlates with invasiveness and aggressiveness in multiple types of cancers^35^. In the present study we investigated the role that miR302 plays in acquired plasticity in primary human mesangial cells. HMCs are significantly affected during renal diseases and these alterations lead to the expansion of mesangial compartment and extracellular matrix deposition^9^,^50^. Our findings demonstrated an unexpected role for the miR302 family as part of a regulatory loop that controls cellular plasticity.

Previous analysis carried out in our laboratory demonstrated that miR302 is upregulated in HMCs undergoing a process of acquired, pro-fibrotic plasticity^4^. This might seem counterintuitive considering that its upregulation is triggered by CTGF stimulation, which is widely accepted to work in concert with TGFβ to activate fibrosis^51^ and impacts directly on the TGFβ pathway. Intrigued by these findings, we investigated the role that miR302 plays in HMC plasticity. During miR302 mediated differentiation, we found persistently high levels of Snail even after a decline in mRNA. In contrast, scramble control cells always exhibited low levels of mRNA and no protein was detectable. This likely reflects the dynamic nature of the reprogramming process where cells are likely to be asynchronous. In addiction, overexpression of miR302 in HMCs causes a strong upregulation of Slug, another member of the Snail family. Snail and Slug are both considered master regulators of the EMT process. This observation is important, since normally miR302 overexpressing cells are prevented from activating Smad3 and Smad2 upon TGFβ stimulation^4^. Nevertheless, increased Snail expression is driven by miR302 in various cell types, consistent across both epithelial and mesenchymal phenotypes (Fig. 1 and Supplementary Fig. S5), pointing toward a TGFβ independent upregulation of the two TFs triggered by miR302. These two transcription factors have been recently implicated in iPSC generation^32^,^52^,^53^. In particular, Snail is required during the initial phase of reprogramming in order for the cells to acquire a higher degree of plasticity, by supressing Let-7 expression^32^, while Slug is pivotal in the later phases, although acting independently of its role as an EMT-transcription factor^52^. Interestingly, miR302 and Let-7 have essentially opposing roles during embryonic development, where miR302 identifies pluripotent stem cells whereas Let-7 is highly expressed in differentiated cells^13^.

EMT involves distinct phenotypic changes through which a polarized epithelial cell gains a scattered mesenchymal phenotype, increasing its motility and invasion properties. A recent paper from the group of Nieto^33^ points out that within injured renal parenchyma, tubular epithelial cells undergo a partial EMT, gaining a hybrid phenotype, in which they acquire Snail expression while maintaining their epithelial hallmarks. According to the authors, this partial EMT of the renal epithelial cells is able to trigger the release of inflammatory and fibrotic cytokines, such as TGFβ, in the microenvironment of the injured kidney leading to the development of fibrosis. Interestingly, HMCs overexpressing miR302 in an *in vitro* healthy context do not become more fibrotic, as indicated by the lack of NFκB expression and TGFβ mRNA. As Snail has been implicated in repressing the expression of Let-7, we analysed the level of expression of Let-7a and c, finding no correlation at early time points post transduction (Fig. 2D). This is perhaps not surprising as the processes of iPSC generation and acquisition of plasticity by HMCs, although having some common ground, remain substantially different. The finding that Smad3 is present and phosphorylated in the nuclei of miR302-HMCs, despite the silencing of the receptor, is intriguing. This suggests that Smad3 is required by cells as an adaptor for phenotypic transition, as its knock down results in the decreased levels of EZH2. Indeed, other TGFβ family members have been proposed to signal in a similar manner; for example Smads likely direct Jmjd3 to target genes in ESCs and during differentiation via direct interaction in response to nodal/activin and facilitating target gene de-repression during endodermal specification^54^. Furthermore, this points to a functional association between the two proteins, even though the expression relationship is not reciprocal as EZH2 knockdown does not affect Smad3 expression. This result supports recent findings from our laboratory that demonstrate a direct interaction between EZH2 and Smad3^46^. Recent work from the Kaji group, demonstrated that the activated forms of Smad3 and Smad2 are not only required during iPSC generation but also promote the direct reprogramming of cells into a different type^55^. Moreover, the use of the SB431542, an inhibitor of the TβRI, during somatic cell reprogramming, enhances the acquisition of pluripotency and in the absence of TGFβ stimulation causes an increase in the level of Smad3/2 phosphorylation, results in line with our observations.

HMCs were found to increase their rate of proliferation with EZH2 overexpression. It has been proposed that EZH2 may play a role independent of its activity in the PRC2 complex^56^. In the present study we confirmed that EZH2 expression is upregulated in miR302-HMCs however the methylation of lysine 27 of histone H3 is decreased (Fig. 3). Interestingly, we recently demonstrated a direct interaction between Smad3 and EZH2 during neuroepithelial differentiation^57^. Silencing of the type II TGFβ receptor by miR302 likely perturbs the interaction with EZH2 while the addition of DZnep erases the H3K27me3 mark, leading to the derepression of epithelial genes as evidenced by *de novo* detection and expression of ZO-1 and E-cadherin in miR302-HMCs. As EZH2 and Snail show the same trend of expression we expected to find a reciprocal interaction between the two of them; however we clearly demonstrated that the lack of EZH2 does not impair Snail expression and vice versa. Nevertheless, these two proteins co-operate to control the acquisition of plasticity in HMCs, as evidenced by *de novo* expression of ZO-1 and E-cadherin, suggestive of MET.

**Figure 7:**
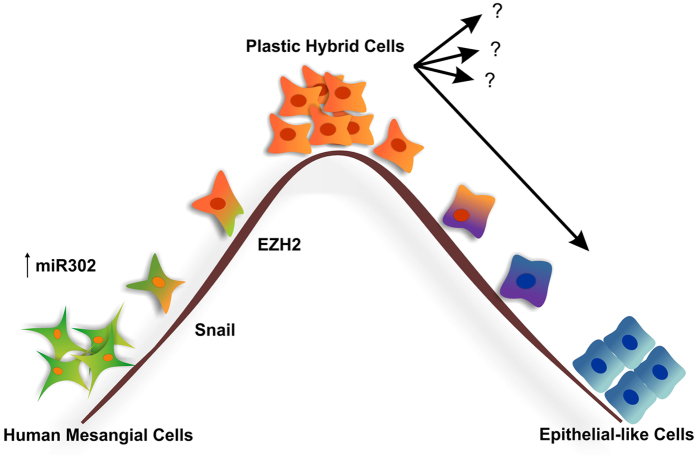
Working hypothesis. Upon miR302 overexpression, HMCs acquired an enhanced plasticity, becoming highly responsive to the surrounding microenvironment (plastic hybrid cells). During this process the cells upregulate the expression of both Snail and EZH2. At this critical tipping point if these cells are placed under the appropriate stimuli, they can acquire epithelial characteristics or, potentially, other kind of phenotypes (question marks). This very dynamic process can be potentially blocked or reversed.

In summary, the complex signalling systems that regulate the acquisition of plasticity in mesangial cells have clear parallels with the processes controlling pluripotency and are likely mediated by a small number of transcriptional master regulators including Smad3 and Snail. Increased miR302 is sufficient to drive cells towards a highly plastic “hybrid” state in which they are susceptible to microenvironmental cues and differentiation. Further understanding of the intricate interplay between TGFβ receptors, Smad3, Snail and EZH2 will not only increase our knowledge of epigenetic and pathogenic processes in diverse diseases such as diabetes and cancer, but will point towards new therapeutic approaches focused on the exploitation and control of cellular plasticity (Fig. 7).

## Material and Methods

### Cell culture, viral transduction, TGFβ and inhibitor treatments of HMCs

Primary HMCs (Human Mesangial Cells; Lonza) were maintained in MCDB-131 medium (Gibco) while Human Kidney Cells (HKC8) were maintained in regular DMEM (Gibco), both supplemented with 10% (v/v) heat-inactivated Fetal Bovine Serum (FBS, Life Technologies), 10 mM L-glutamic acid (Gibco) and 100 U/ml penicillin and 100 μg/ml streptomycin (Gibco). For TGFβ stimulation, HMCs and HKC8 were serum starved for 24 h. Recombinant human TGFβ1 (Promokine) was used at a final concentration of 5 ng/ml and cells were stimulated for 24 h, 48 h and 72 h. Protein and RNA were harvested at each time points. 3-Deazaneplanocin A (DZNep, Calbiochem) was added 1 h before TGFβ at a final concentration of 5 μM.

For viral transduction, HMCs and HKC8 were seeded at 5 × 10^4^ cells/well. The following day cells were transduced with a lentivirus carrying the human miR302a/b/c/d cluster (System Biosciences) or a scramble sequence (pGiPz). The media was changed 48 h post transduction and replaced with DMEM-F12 (Sigma) supplemented with 10% v/v FBS, 10 Mm L-glutamine, 100 U/ml penicillin and 100 μg/ml streptomycin. The successfully transduction was verified through GFP expression. Non-transduced HMCs and HKC8 maintained in DMEM-F12 were used as a positive control.

The treatment with the SB431542 (Calbiochem) compound was carried out at 7 days post transduction for either 7 days (day 14 post transduction) or 14 days (day 21 post transduction) in mir302-HMCs scramble infected HMCs and non-treated HMCs. The final concentration was 10 μM, media was changed every other day, no splitting was performed during the inhibitor treatment. Protein and RNA were extracted at day 7 and 14 post treatment.

### Differentiation protocol

7 days after viral transduction miR302 and scrambled-infected HMCs were adapted to KnockOut™ Serum Replacement (Thermo Fisher Scientific) media for 5 days. At day 13 post-transduction the cells were trypsinized and plated on matrigel in the following media: DMEM-F12, 20% KnockOut™ Serum Replacement, 0.01 mM non-essential amino acid (NEAA, Gibco), 100 U/ml penicillin, 100 μg/ml streptomycin, 0.05 mM 2-mercaptoethanol (Sigma), 25-ng/ml bFGF (BioLegends) plus and minus 0.1 μM DZNep. The cells were maintained in culture for up to a week.

### Matrigel coating

Growth-factor reduced matrigel (BD Bioscience) was prepared by diluting the matrigel in a ratio of 1:10 with cold serum-free media. The matrigel was washed three times in the plate and then the plate was incubated with the lid off for 30 minutes at room temperature inside the laminar hood. The plate was then incubated at 37 °C for 1 hour and washed one time with serum-free media before cells were seeded onto the plate.

### Picking and culturing of HMC-colonies

After their initial appearance, colonies morphologically resembling ES colonies were picked and subcultured. Briefly, individual colonies were subcultured on matrigel in ES cell medium comprising Murine Embryonic Fibroblasts (MEFs) conditioned media, 20% KnockOut™ Serum Replacement, 0.01 mM non-essential amino acid, 100 U/ml penicillin, 100 μg/ml streptomycin, 0.05 mM 2-mercaptoethanol, 25-ng/ml bFGF. The colonies were cultured for up to 21 days.

### Analysis of mRNA expression

Total RNA was extracted from HMCs at days 3, 7, 14, 21 and 28 post-transduction according to the manufacturer’ s instruction (TRIzol®, Life Technologies). cDNA was synthesized starting from 500 ng of RNA as previously described^58^. Quantitative RealTime PCR was performed using TaqMan assays for TβRII, Snail and CDH1. Data was normalized using 18 S as endogenous control.

### microRNA analysis

Total RNA was extracted from HMCs at day 7 post-transduction according to the manufacturer’s instruction (TRIzol®, Life Technologies). cDNA was synthesized using TaqMan qRT-PCR assay for miR302 d, Let-7c and Let-7a. Data was normalized using RNU6B as endogenous control.

### Western Blot analysis and nuclear-cytoplasmic fractionation

Total protein was isolated from HMCs at day 3, 7, 14, 21 and 28 using RIPA buffer (Tris-HCL, NaCl, NP-40, Na-deoxycholate, and Ethylenediaminetetraacetic acid (EDTA)) supplemented with 1 mM NaF, 1 mM Na_2_VO_4_, 50 mM phenylmethylsulphonylfluoride (PMSF) and protease and phosphatase inhibitor cocktails (Sigma). Nuclear-cytoplasmic fractionation of HMCs was achieved by suspending the cells in an isotonic buffer containing 10 mM HEPES, 1.5 mM MgCl2, 10 mM KCl, 0.5 mM Dithiothreitol (DTT) and protease inhibitor cocktails, left on ice for 15 min to separate the nuclei from the cytoplasm and then centrifuged for 1 min at 12,000 × g at 4 °C. Nuclear pellets were then resuspended in a solution containing 20 mM HEPES, 25% v/v Glycerol, 0.42 M NaCl, 1.5 mM MgCl2, 0.2 mM EDTA, 05 mM PMSF, 0.5 mM DTT and protease inhibitor. The solution was then incubated for 30 min on a shaker at 4 °C and subsequently centrifuged. Total protein concentration was determined by Bradford Assay (Biorad). The following primary antibodies were used: Snail, Slug, TβRII, GAPDH, NFκB, caspase 3, Smad3/P (all from Cell Signalling Technologies), E-Cadherin (BD pharmingen), EZH2 (kind gift from the Bracken Laboratory, Trinity College Dublin, Ireland), SUZ12 (Cell Signalling), p53 (Calbiochem), β-actin (Sigma), Histone 3 and H3K27 (all from Abcam).

### Immunofluorescence staining

HMCs were fixed with 4% paraformaldehyde for 10 minutes, followed by 1 h incubation at room temperature with rabbit polyclonal anti-ZO-1 (Invitrogen) primary antibody diluted in 5% Goat Serum (Sigma). Goat anti-rabbit Texas Red (Invitrogen) was used as secondary antibody and incubated for 1 h at room temperature. Nuclei were counterstained with DAPI (4,6 diamidino-2-phenylindole, Sigma).

### Proliferation and apoptosis assay

At day 7 and 21 post-transduction, HMCs were seeded onto a 96-well plate at a concentration of 500 cells/well. Non-transduced HMCs were seeded at the same concentration, as a control. 10 μl of MTT (3-(4,5-Dimethylthiazol-2-yl)-2,5-Diphenyltetrazolium Bromide, Sigma) diluted in ddH_2_O (5 mg/ml) was added to each well, and the plate was incubated at 37 °C for 4 hours. Following incubation, 100 μl of dimethyl sulphoxide (DMSO, Sigma) was added to each well. Optical Density (OD) was read at 570 nm and 650 nm with an ELISA reader (Molecular Devices). In one set of experiments cells were treated at the time of the seeding with 5 μM DZnep.

To trigger apoptosis, HMCs were incubated with 50 μM of etoposide (Merck) for 24 h as follow. At day 7 post transduction miR302, scramble and non-transduced cells were seeded at confluency of 50%. The day after the cells were gently washed with PBS (Sigma) and fresh media was added along with 50 μM of etoposide. The cells were lysed in RIPA buffer after 24 h.

### Gene Knock down using the esiRNA technology

Endoribonuclease-prepared siRNA (esiRNA) were prepared as described by Heninger and Buchholz^59^, with a few modifications. In brief, cDNA was isolated from NT2 cells and used as a template from which to generate T7 RNA polymerase promoter-flanked cDNA fragments, through two rounds of PCR amplification. These fragments represented the region of mRNA against which the esiRNAs would target genes of interest. These fragments were subsequently transcribed *in vitro* to generate double-stranded RNA (dsRNA) of the target amplicons, which were purified to isolate only larger (>250 bp) RNA molecules. Following purification, dsRNA was digested using a Ribonuclease III (RNase III) mutant with a single amino acid substitution (E38A), which was produced in *Escherichia coli*^60^ to generate a heterozygous population of ~21 nt siRNAs, capable of targeting multiple regions of target mRNA. Post-digestion, the siRNA fragments were purified using a QIAGEN RNeasy kit using high ethanol concentrations. See table 1 for a complete list of primers.

miR302, scramble (Firefly luciferase) and non-transduced HMCs as control, were transfected using Lipofectamine® 2000 (Invitrogen) according to manufacturer’s instruction 7 days post viral transduction. 48 h after transfection the cells were lysate for protein extraction.

### Statistical analysis

The reported values were represented as the mean ± standard error of mean. One-way Anova and Bonferroni post-test were used to calculate the statistical significance for the analysis of more than two categories, Student’s t-test was used in the charts were only 2 categories are presented (*P < 0.05, **P < 0.01, ***P < 0.001, ****P < 0.0001). All the analyses were performed with PRISM5.02 (GraphPad Software Inc, La Jolla CA, USA).

## Additional Information

**How to cite this article**: De Chiara, L. *et al*. miR302 regulates SNAI1 expression to control mesangial cell plasticity. *Sci. Rep.* **7**, 42407; doi: 10.1038/srep42407 (2017).

**Publisher's note:** Springer Nature remains neutral with regard to jurisdictional claims in published maps and institutional affiliations.

## Supplementary Material

Supplementary Data

## Figures and Tables

**Table 1:** Primers sequences for esiRNAs synthesis.

Gene name	Primer F (target gene only)	Primer R (target gene only)
SNAI1	TTTACCTTCCAGCAGCCCTA	CCAGGCTGAGGTATTCCTTG
EZH2	GAGGACGGCTTCCCAATAAC	GGAGCTGGAGCTATGATGCTA
FFlucGL3	CGGATTACCAGGGATTTC	CCTCAGAAACAGCTCTTC
SMAD3	ACAAGGTCCTCACCCAGATG	TGGACTGTGACATCCCAGAA
