# Corrigendum: miR302 regulates SNAI1 expression to control mesangial cell plasticity

**DOI:** 10.1038/srep46802

**Published:** 2017-02-14

**Authors:** Letizia De Chiara, Darrell Andrews, Ariane Watson, Giorgio Oliviero, Gerard Cagney, John Crean

Scientific Reports 7: Article number: 4240710.1038/srep42407; published online: 02 14 2017; updated: 04 09 2017

The original version of this Article contained errors in the spelling of the authors Letizia De Chiara, Darrell Andrews, Ariane Watson, Giorgio Oliviero, Gerard Cagney and John Crean which were incorrectly given as L. De Chiara, D. Andrews, A. Watson, G. Oliviero, G. Cagney and J. Crean.

This has now been corrected in the PDF and HTML versions of the Article.

