# Supplementary material for: miR302 regulates SNAI1 expression to control mesangial cell plasticity
Source: Sci Rep. 2017 Feb 14;7:42407. doi: 10.1038/srep42407 (PMC5307964; doi:10.1038/srep42407)
Supplement: Supplementary Data [file srep42407-s1.pdf]

miR302 regulates SNAI1 expression to control mesangial cell plasticity

De Chiara, L.*, Andrews, D., Watson. A., Oliviero, G., Cagney, G. and Crean, J.

SUPPLEMENTARY INFORMATION

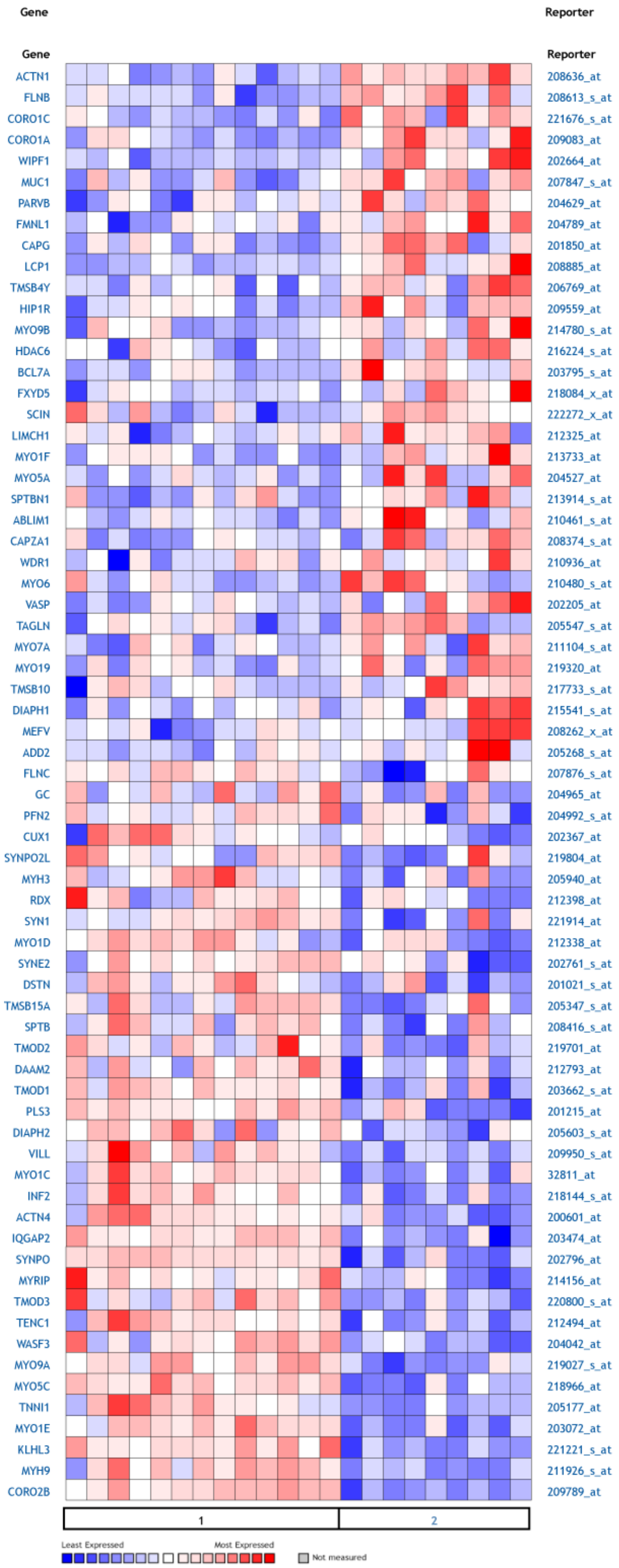

Supplementary Figure S1. Differential gene expression in diabetic nephropathy is evidence of mesangial plasticity. Samples from the glomeruli of healthy living donors (n=12) and diabetic nephropathy patients (n=10) were taken and subjected to microarray analysis (Affymetrix Human Genome U133A). Nephroseq (The Regents of The University of Michigan, Ann Arbor, MI) was used for analysis and visualization. Actin binding and regulatory genes were extracted by gene ontology (GO) search. (p value < 0.05). Dynamic remodelling of the actin cytoskeleton during the pathogenesis of disease is widely accepted to reflect reactivation of embryogenic, plastic processes in adult and mature mesangial cells.

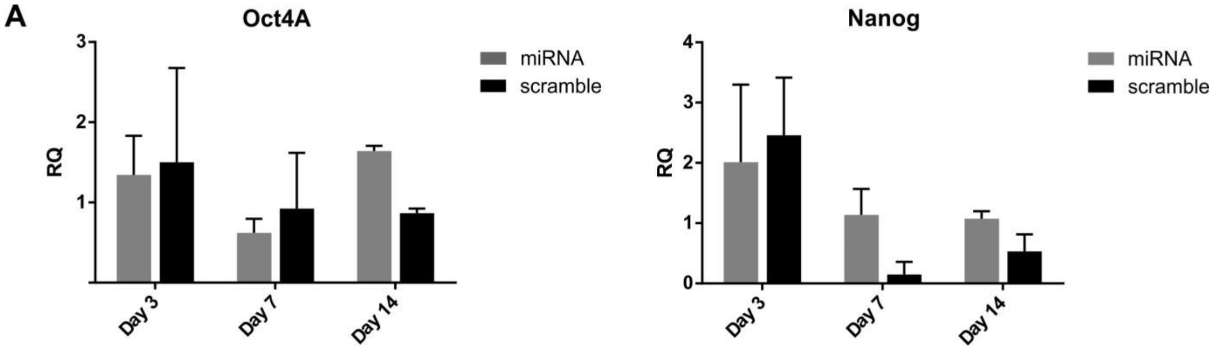

Supplementary Figure S2. miR302-HMCs do not acquire pluripotency. RNA was extracted at different time points and the expression of pluripotency markers was tested (A). No change in the expression of Oct4A and Nanog was detected at all the analysed time points. Data are representative of 3 independent experiments.

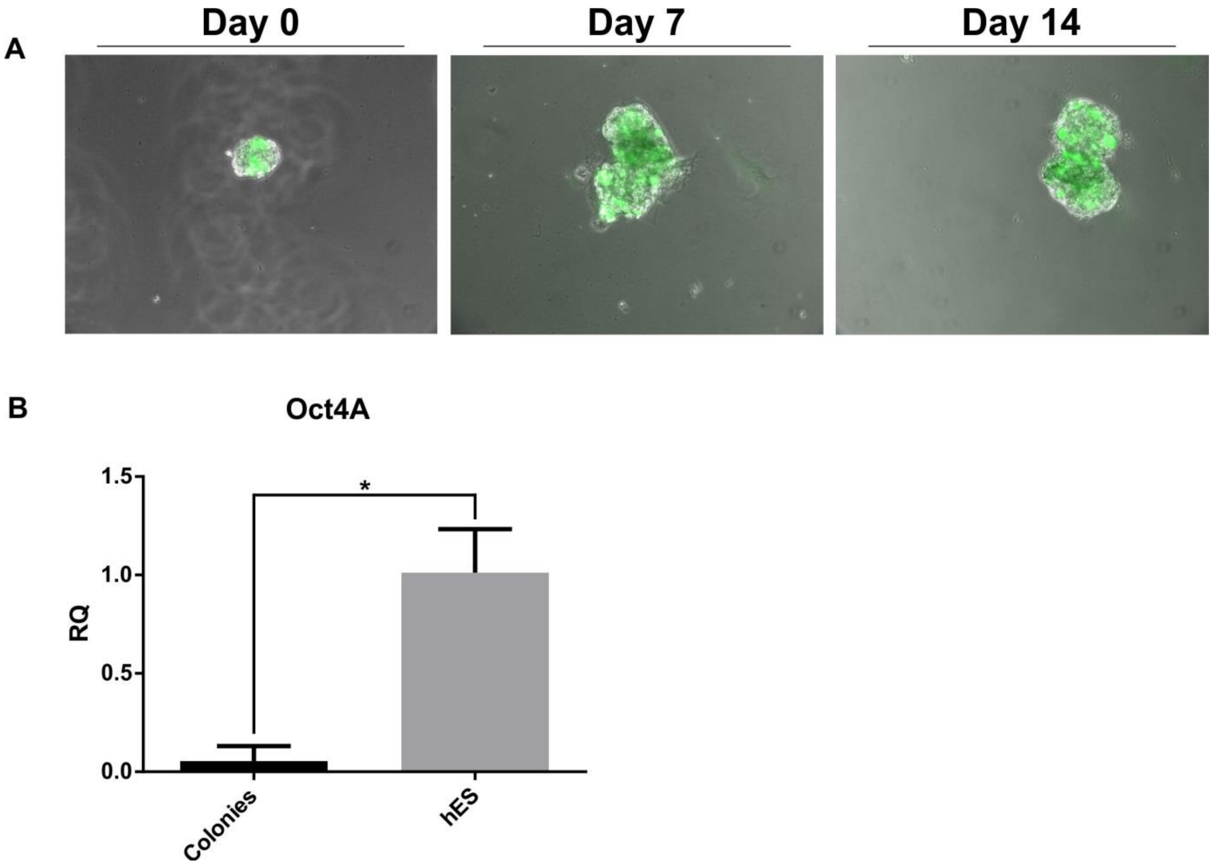

Supplementary Figure S3. Colony formation of miR302-HMCs. Rounded colonies started to appear in miR302-HMCs between 17 and 21 days in culture (A, left panel). After picking, these colonies were cultured in stem-like culture condition on matrigel for up to 14 days. 7 days post picking the colonies expanded and became bigger (middle panel) but they were unable to separate (right panel). Colonies were analysed for Oct4A expression 14 days after picking however none was detectable (B), indicating their non-pluripotent nature. Original magnification x100. (* $P < 0.05$). Human Embryonic Stem Cells (hESC) RNA was used as control (kind gift of Oliviero' s lab HuGeF, University of Turin).

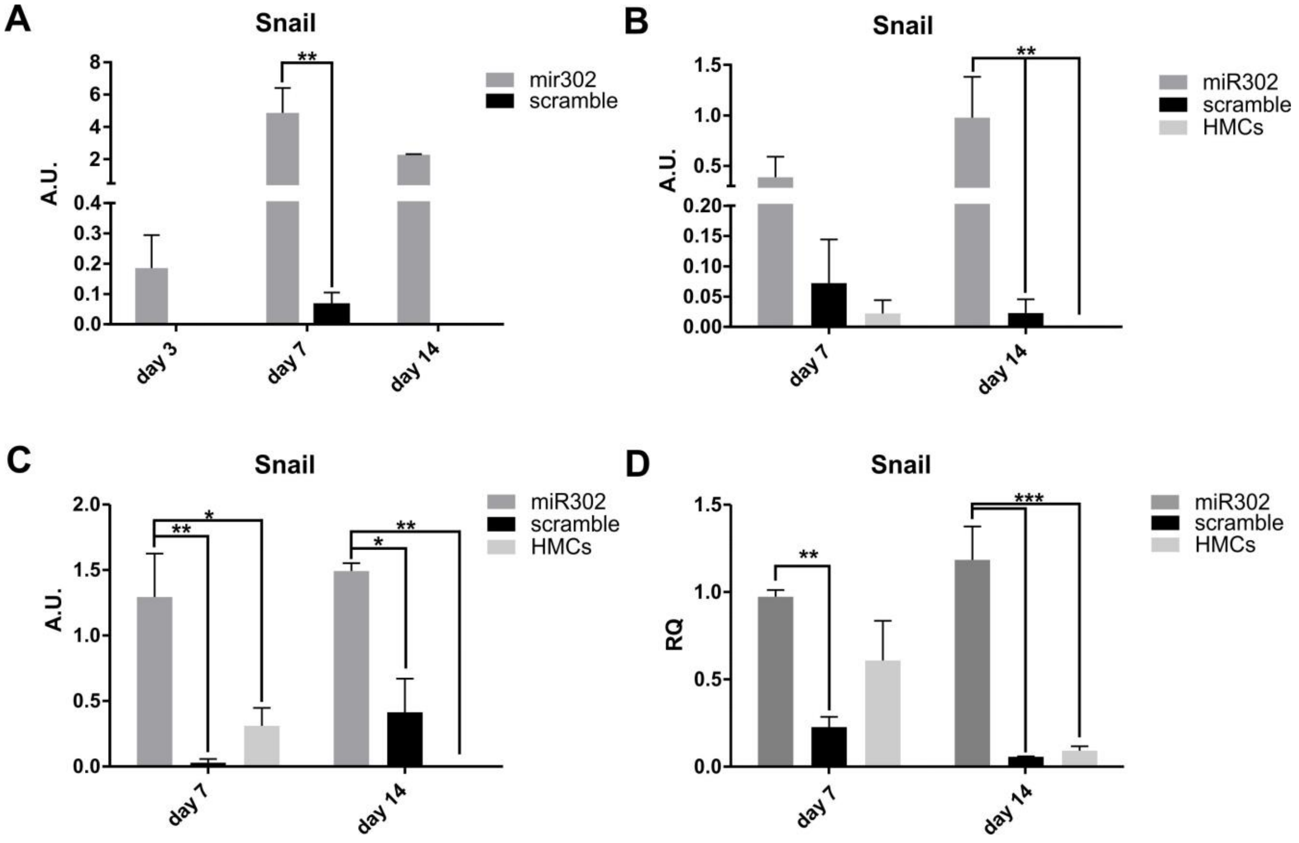

Supplementary Figure S4. Quantification of Snail expression in transduced HMCs. Densitometric analysis of N=3 experiments, normalized to either GAPDH (A) or β-Actin (B) and (C). Figure (A) shows Snail upregulation at all the analysed time points in miR302-HMCs compared to scramble infected HMCs. Snail upregulation becomes statistically relevant at day 7. Figures B and C represent the quantification of Snail expression in miR302 and scramble HMCs respectively with (B) or without (C) treatment with the SB431542, a potent TGFβ inhibitor. Panel (D) represents RealTime analysis of Snail 7 days and 14 days of treatment with the SB431542. (* $P < 0.05$, ** $P < 0.01$), (A.U.: Arbitrary Unit). Data are representative of 3 independent experiments.

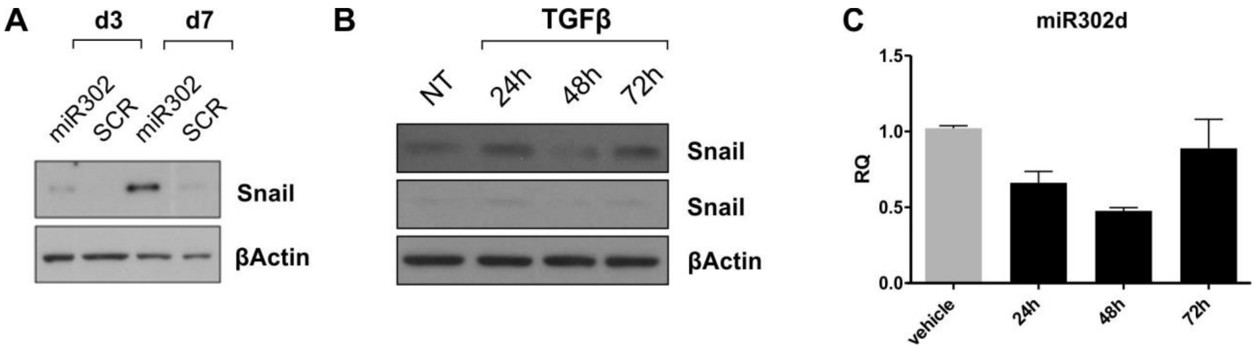

Supplementary Figure S5. TGFβ and miR302 show opposing effects and in HMCs. As in HMCs, the renal epithelial cell line HKC8 showed increased overexpression of Snail (A) at 7 days post miR302-transduction. TGFβ treatment do not alter the expression of Snail in HMCs (B). Top and bottom panel represent the same gel at two different exposure times. Interestingly miR302 is reduced in HMCs upon TGFβ stimulation (C). (SCR: Scramble; NT: non-treated). The panels represent 3 independent experiments.

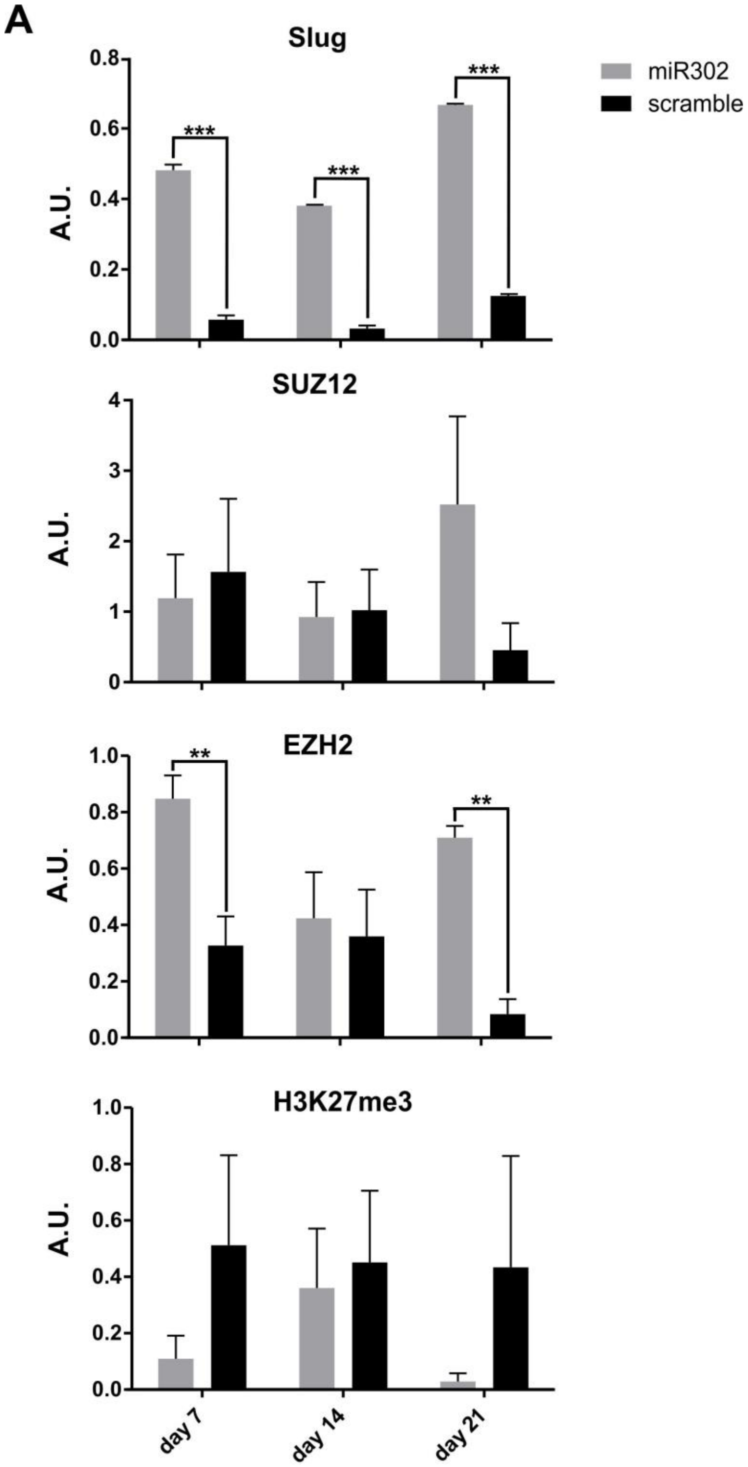

Supplementary Figure S6. miR302 causes upregulation of both Slug and EZH2. Densitometric analysis of N=3 experiments, normalized to β-Actin. The graphs show a positive upregulation of both Slug and EZH2 following miR302 overexpression, while no difference is shown for SUZ12, another component of the PRC2 complex. The trimethylation mark on the lysine 27 of the histone H3 is higher in scramble HMCs at day 7 compared to miR302-HMCs. (* $P < 0.05$, ** $P < 0.01$), (A.U.: Arbitrary Unit). Data are representative of 3 independent experiments.

A

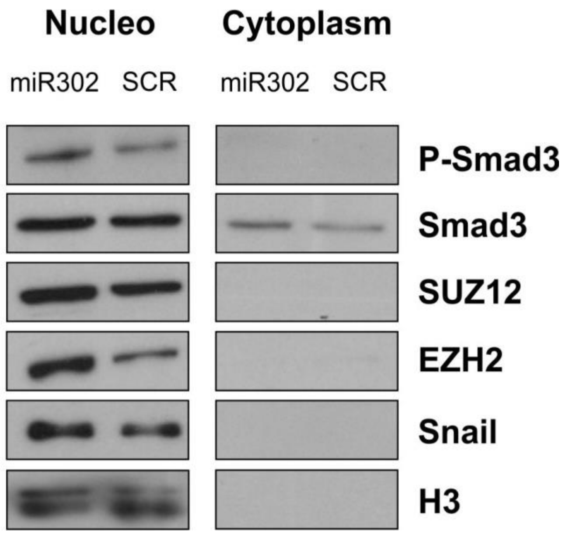

Supplementary Figure S7. miR302-HMCs maintain expression of Smad3. Fractionated extracts prepared from day 7 transduced cells (A) confirmed the correct localization of all the analysed protein. Surprisingly Smad3 is not depleted in the nuclear fraction indicating a possible role for Smad3 in mediating plasticity. (SCR: Scramble). The panel represents 3 independent experiments.

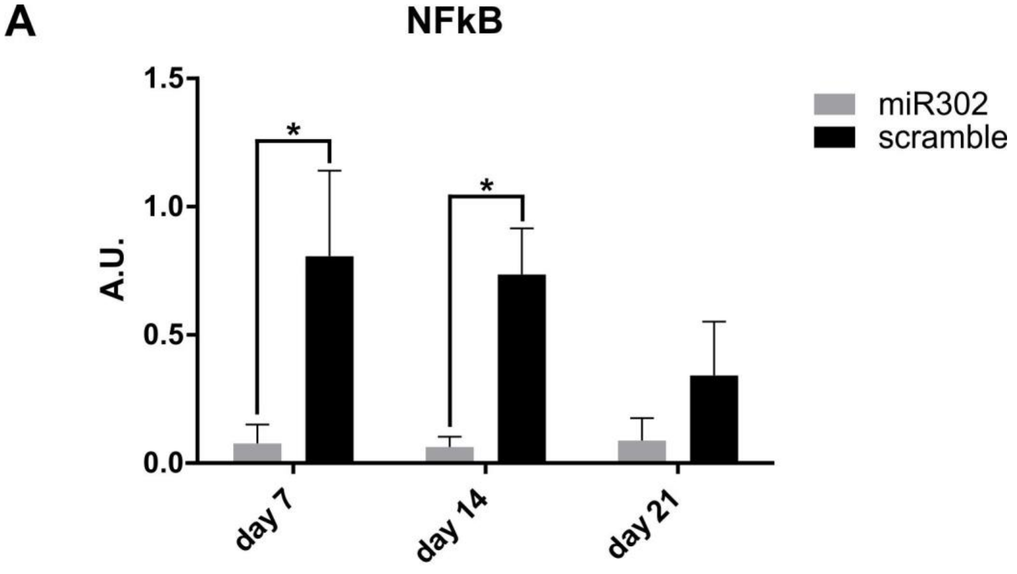

Supplementary Figure S8. Quantification of NFκB expression. Densitometric analysis of N=3 experiments, normalized to β-Actin. (* $P < 0.05$, ** $P < 0.01$), (A.U.: Arbitrary Unit).

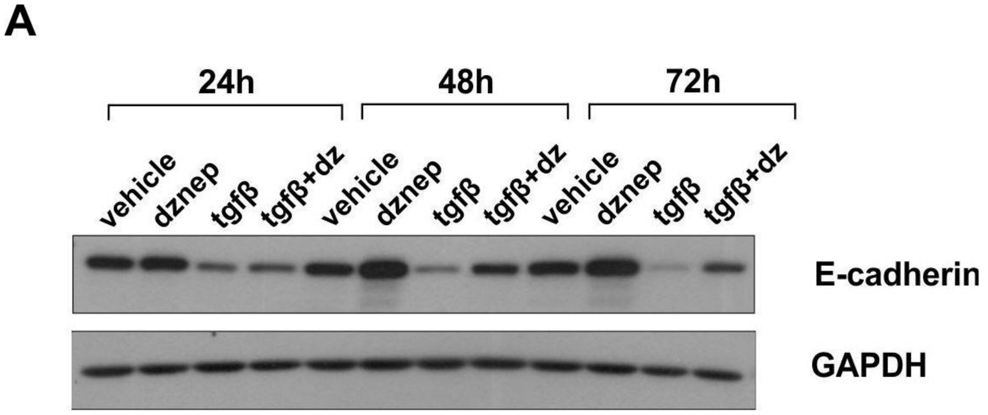

Supplementary Figure S9. DZnep treatment increases the level of E-cadherin in renal epithelial cells. HKC8 were treated for up to 72h with DZnep (A) to prevent cells from undergoing EMT. Interestingly, the control cells (DZnep only) showed an increased level of E-cadherin protein expression when compared to the vehicle treated cells.
